# Introduction and implementation of an immunization information system in the Indonesian province of Daerah Istimewa Yogyakarta: lessons for scaling-up

**DOI:** 10.1186/s12913-022-08910-6

**Published:** 2023-01-05

**Authors:** Sulistyawati Sulistyawati, Trisno Agung Wibowo, Rokhmayanti Rokhmayanti, Andri Setyo Dwi Nugroho, Tri Wahyuni Sukesi, Siti Kurnia Widi Hastuti, Surahma Asti Mulasari, Marta Feletto

**Affiliations:** 1 Faculty of Public Health, Universitas Ahmad Dahlan, Kampus 3 - Jl. Prof Dr Soepomo, Janturan, Umbulharjo, Yogyakarta, Indonesia; 2 Daerah Istimewa Yogyakarta (DIY) Health Office, Yogyakarta, Indonesia; 3 Alliance for Health Policy and Systems Research, World Health Organization, Geneva, Switzerland

**Keywords:** Immunization, Electronic immunization registry, Immunization information system, Interoperability, Implementation research

## Abstract

**Background:**

Immunization is critical to saving children from infections. To increase vaccination coverage, valid and real-time data are needed. Accordingly, it is essential to have a good report system that serves as defaulter tracking to prevent children's immunization failure. The Daerah Istimewa Yogyakarta (DIY) Health Office introduced an electronic immunization registry and successfully implemented it for more than five years. It is the only individual-based record system in Indonesia that has been sustainably operated for a long time. Yet, no systematic assessment of this system has been conducted to date. This study examines the Sistem Informasi Imunisasi Terpadu (SIMUNDU) introduction and implementation process with a view to extracting lessons that could inform scalability and sustainability across the country.

**Methods:**

This study used an explanatory sequential mixed-method design, which collected quantitative data from 142 participants and qualitative data from nine participants. The data entry clerk at a health facility was systematically selected to participate in the survey, while in the key informant interview, the informant was selected based on the survey result. A descriptive and thematic approach was adopted to analyze the quantitative and qualitative data. Results from across the two approaches were integrated for comparison and contrast.

**Results:**

Findings are presented according to three core themes that emerged from the data: system strengths, potential threats, weakness and opportunities for scaling-up. Strengths, i.e., factors contributing to the success of SIMUNDU, include management, system performance, people’s behavior, and resources. Potential threats to sustaining the system include individual capacity, technical or system issues, and high workload. Opportunities – i.e., a promising factor that influences the SIMUNDU ability to operate sustainably – such as continuity, expectation, and the possibility of scaling up.

**Conclusions:**

SIMUNDU is a promising innovation for Indonesia, beyond DIY. There is agreement about the potential for scaling up this IIS to other provinces. The experience of implementing this system in DIY over the past five years has shown that the benefits outweigh the challenges, and SIMUNDU has grown into a robust yet user-friendly system.

**Supplementary Information:**

The online version contains supplementary material available at 10.1186/s12913-022-08910-6.

## Background

Neonatal and childhood vaccination is essential for infectious disease prevention and an absolute human right [1, 2]. Vaccination has been proven to reduce the burden of infectious diseases globally [3]. According to the WHO, in 2020, an estimated 23 million children under the age of one year did not receive their essential vaccinations. Of these, 60% live in just ten countries, one of which is Indonesia [4]. Indonesia is the fourth most populous country globally. It is composed of thousands of islands organized into 34 provinces. Various geographical and cultural factors influence population inequalities in accessing health services [5]. In 2001, the Indonesian government's decentralization policy was enacted. This was an excellent strategy for fostering development by engaging regional resources [6]. However, this strategy was not without consequence. One primary concern was the health information system (HIS) fragmentation.

Indonesia's federal structure results in provinces and districts being relatively independent of the national Ministry of Health. This means that provincial- and district-level information systems are locally regulated [7]. For instance, *Pemantauan Wilayah Setempat* (PWS) is a management tool used to monitor the coverage of specific health services within an administrative boundary. Depending on the service and region, it can be paper- or electronic-based. PWS-KIA is the monitoring system specific to maternal and child health (KIA), including immunization. PWS-KIA data are reported to the District or City Health Office, go to the Province Health Office, and are finally reported to the main level. Generally, the data are in Microsoft Excel formats; they will be reported via emails or various information systems, including Komdat Kesmas, SITT, SIHA, PISPK, and SIKDA Generik. PWS-KIA data feed into District Health Information System 2 (DHIS2). Regional information systems have varying data quality, which reflects inequities in resources across regions. This adds to data integration challenges at the national level [7, 8] and affects strategic policymaking.

In Indonesia’s federal system, Daerah Istimewa Yogyakarta (DIY) province has the authority to regulate and use its budget within its four districts plus one city (Sleman, Gunungkidul, Bantul, Kulonprogo, and Yogyakarta). This province is classified as a small province in terms of area size and the number of regions inside [9]. However, this region can be considered a representation of Indonesia when viewed from the geographical, socioeconomic, and heterogeneous population perspective. With regard to childhood vaccination, DIY is among the top ten performing provinces in the country, with 97.7% of children completing basic immunization coverage in 2019 [10]. Immunization services are provided by primary health centers or Puskesmas (PHC), as well as private clinics, hospitals, and midwives' practices (typically referred to as *Unit Pelayanan Swasta* or UPS).

An electronic immunization registry is a tool for recording individual children’s immunization histories. In 2014, the DIY Health Office introduced an electronic immunization registry named SIMUNDU (*Sistem Informasi Imunisasi Terpadu*/ Integrated Immunization Information System). An electronic registry provides essential functions at all levels of the health system. At the district and higher levels, it allows for monitoring vaccination coverage by vaccine, dose, cohort, and other variables – and can support microplanning and vaccine management. The service delivery level can facilitate individual follow-up of vaccination status and enable health workers to identify children due for vaccination and those who have missed their vaccinations (defaulters).

SIMUNDU was designed to link with PWS-KIA for immunization and interoperability with the DHIS2. While it predominantly contains individual-level immunization records, SIMUNDU also serves as a source for aggregation and can synergize with the *Pemantauan Wilayah Setempat* (PWS) reporting system. For this reason, it can be considered an immunization information system (IIS). This means that city and district levels feed into provincial and national levels *(Personal communication with DIY immunization program officer)*.

**Fig. 1:**
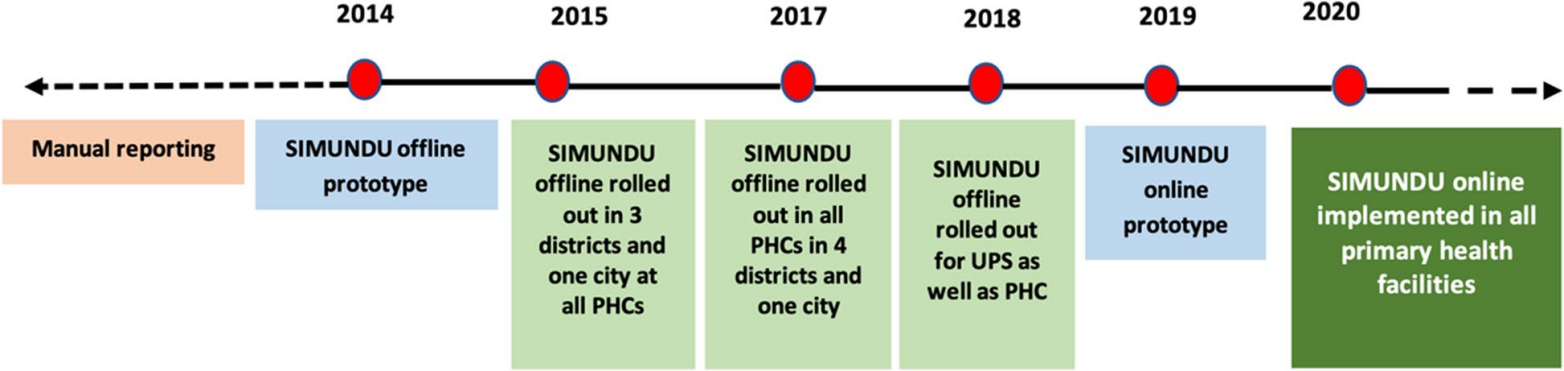
SIMUNDU’s development and introduction

The original prototype was designed by the information and technology (IT) department of the DIY Health Office to be operated offline. In DIY, three out of the four districts and the city introduced the system in 2015. The final district introduced it in 2017. At this stage, the point of data entry was the PHC only. By 2018, UPS facilities were also equipped with SIMUNDU and could enter data into the system. In 2019, the prototype was further developed to operate online. The online version was rolled out in 2020 (Fig. 1). As of May 2021, 79.4% of all PHC and UPS facilities complied. This average rate masks, however, the fact that while all PHCs adopt SIMUNDU, it is more challenging to enforce its use in UPC facilities (Suyani 2020, oral communication, 2020, May 11).

When a child receives a vaccination in a health facility, information on the child and the vaccination is entered in SIMUNDU as an individual child record. Each record includes a personal identifier, the child’s sociodemographic characteristics (e.g., name, gender, date of birth, name of parents, address), the antigen administered, and the date and place of vaccination. SIMUNDU has been recently updated to allow the recording of vaccinations administered in schools (e.g., human papillomavirus (HPV), diphtheria toxoid (DT), tetanus-diphtheria (TD), and measles-rubella (MR)), albeit in the form of aggregate data only. Furthermore, SIMUNDU has been developed to record COVID-19 vaccinations in health facilities and those carried out en masse.

Monitoring is conducted monthly to assess data completeness across health facilities, while an evaluation is conducted yearly. These exercises have allowed the identification of several challenges related to implementing the system (e.g., workload, staff turnover, and rotation) and data quality (e.g., accuracy and timeliness). However, no systematic assessment of the system has been conducted to date. SIMUNDU is the first immunization information system ever introduced in Indonesia. Other districts and provinces have shown interest in rolling it out, and the Ministry of Health has acknowledged the innovation. The work presented here aims to examine SIMUNDU’s introduction and implementation process with a view to extracting lessons that could inform scalability and sustainability across the country.

## Methods

From May to October 2020, we examined the experience of introducing and implementing an immunization information system in DIY province using an explanatory ﻿sequential mixed-method design, where each step informed the next [11]. First, we reviewed all relevant documentation available in the DIY Health Office – e.g., staff notes, meeting notes, and monitoring notes – documenting SIMUNDU development and management processes. We also examined online documents, including health profiles and regulations on health-reporting systems in Indonesia. This served as the initial data source and provided an overview of who was involved and how in developing and implementing SIMUNDU. This informed the survey design that we conducted as a second step. The survey targeted any staff responsible for entering data in SIMUNDU (i.e., data clerks) across all PHC and selected UPS facilities and any staff responsible for managing the system at the district and city level (i.e., immunization coordinators). Sampling and recruitment strategies are outlined in Table 1.

**Table 1:** Survey participants

Level of the data entry and reporting system	Total number of facilities/ offices	Study population	Sampling strategy	Recruitment	Sample size
Primary Health Center (PHC)	121	Data entry clerks	All facilities	Open invitation across all facilities	113
UPS – Central, General, Maternity, and Pediatric Hospitals	65	Data entry clerks	Randomly selected 2 facilities per district/city (2*5 = 10)	Open invitation across selected facilities	8
UPS – Clinics	73	Data entry clerks	Randomly selected 2 facilities per district/city (2*5 = 10)	Open invitation across selected facilities	7
UPS – Midwives’ Practices	271	Data entry clerks	Randomly selected 2 facilities per district/city (2*5 = 10)	Open invitation across selected facilities	10
District/City Health Office	5	Immunization coordinators	Total sampling	Open invitation	4*
Total					**142**

^*^When the immunization coordinator recently changed, the former was also invited

All immunization coordinators in each district/city and data entry clerks from all primary health facilities (PHCs) were invited to participate in this survey. For UPS facilities, we selected two clinics, two midwives’ practices, and two hospitals per district/city and invited all of their staff who were involved in SIMUNDU data entry and management.

We developed and pretested an online survey in Bahasa Indonesia to inquire about SIMUNDU implementation, processes, and outcomes across PHC, UPS clinics, and district or city and province offices. The questionnaire consisted of closed-ended and Likert scale questions – ranging from 45 to 50 depending on the target type of facility and/or level of the health system – and enquired about respondents’ sociodemographic characteristics as well as the process of implementing and managing SIMUNDU. Some questions provided an additional field for clarifying the reason for a particular answer choice.

All participants were invited to the DIY Health Office to complete the survey on their laptops, with their prior consent. All participants in a room allowed researchers to monitor any missing or incomplete responses in real time and follow up with individual participants on-site to fill any gaps. We don’t believe this may have introduced any significant bias as researchers would simply flag any missing responses and invite respondents to address those. Data were then exported and analyzed in Microsoft Excel. The topic areas for the qualitative interview were informed by an exploratory analysis of the survey data.

Similarly, some informants were purposefully selected among survey participants to follow up on the range of perspectives that had emerged from the survey. Other informants had been identified at the desk review stage and chosen for their management functions. Selected informants were invited to the DIY Health Office for the interview, and COVID-19 prevention protocols were observed. Every informant was informed about the study and asked to sign the informed consent. All invited informants agreed to participate. A total of nine 30-min semi-structured interviews were conducted in the Bahasa Indonesia language and recorded with prior consent from participants. The interview team consisted of three researchers with the respective tasks of running the interview, observing, and taking notes. A research assistant transcribed all interviews into Bahasa Indonesia.

Thematic analysis was conducted using the Quirkos qualitative tool following Braun and Clarke’s approaches [12]. Researchers familiarized themselves with the data, searching for initial codes and allowing themes to emerge. The principal investigator led the coding process, and led the research team too in defining and naming the core themes emerging from the data, organizing and analyzing the data across the themes, and triangulating information from the desk review, the survey, and the interviews. This stage was also performed in Bahasa Indonesia. Data were translated into English only at subtheme and core themes levels’.

## Results

### Participant characteristics

#### Quantitative study

In total, 142 respondents participated in this study spread across five districts/cities in DIY province. Among them, Gunungkidul has a higher proportion of respondents than the other district, with 24.8%, 24%, and 25% for PHC, UPS, and DHO, respectively. For all research units, the majority were women. At the UPS and DHO/CHO levels, most respondents were aged 41–45 years, i.e., 28.3% and 75%, respectively, while at the UPS level, the majority were aged 25–30 years (56.0%). In terms of education level, PHC and UPS are dominated by Diploma 3 graduates, namely 86.7% and 80%, respectively, while in DHO/CHO, there are predominantly undergraduate graduates (75%) (Table 2).

**Table 2:** Characteristic participants in three groups of respondents

Characteristic	PHC (*n* = 113) n (%)	UPS (*n* = 25) n (%)	DHO/CHO (*n* = 4) n (%)
District/City			
Bantul	23 (20.4)	5 (20.0)	1 (25.0)
Gunungkidul	28 (24.8)	6 (24.0)	1 (25.0)
Yogyakarta	17 (15.0)	4 (16.0)	0 (0.0)
Kulonprogo	21 (18.6)	4 (16.0)	1 (25.0)
Sleman	24 (21.2)	6 (24.0)	1 (25.0)
Sex			
Male	3333(2.7)	0 (0.0)	2 (50.0)
Female	110 (97.3)	25 (100)	2 (50.0)
Age			
< 25	0 (0.0)	5 (20.0)	0 (0.0)
25–30	3 (2.7)	14 (56.0)	0 (0.0)
31–35	30 (26,5)	3 (12.0)	0 (0.0)
36–40	19 (16.8)	1 (4.0)	0 (0.0)
41–45	32 (28.3)	0 (0.0)	3 (75.0)
46–50	18 (15.9)	0 (0.0)	1 (25.0)
> 50	11 (9.7)	2 (8.0)	0 (0.0)
Education			
Master	0 (0.0)	1 (4.0)	1 (25.0)
Bachelor	5 (4.4)	1 (4.0)	3 (75.0)
Diploma 4	9 (8.0)	2 (8.0)	0 (0.0)
Diploma 3	98 (86.7)	20 (80.0)	0 (0.0)
Senior high school	1 (0.9)	1 (4.0)	0 (0.0)

#### Qualitative study

Nine informants were recruited to provide the required information to explore the quantitative study results more deeply. They serve as managers and staff at DHO/CHO, PHC, and UPS. Among the nine informants, two were men and seven were women. Three informants graduated with a master’s, one with a bachelor's, and there were five graduates with diplomas (Table 3).

**Table 3:** Informants’ characteristics for the qualitative study

Sex	Age (years)	Education	Position	Subject group	Informant’s code
Female	56	Master’s	Head of disease prevention and control department at PHO level	Managerial	M 01
Male	57	Master’s	The former head of the disease prevention and control section at the PHO level	Managerial	M 02
Male	54	Bachelor’s	Immunization programmer (coordinator) at the PHO level	Managerial	M 03
Female	47	Master’s	IT person	Managerial	M 04
Female	34	Diploma	Data entry at the PHC level	Staff	S 01
Female	25	Diploma	Data entry at the UPS level	Staff	S 02
Female	31	Diploma	Data entry at the UPS level	Staff	S 03
Female	42	Diploma	Data entry at the PHC level	Staff	S 04
Female	24	Diploma	Data entry at the PHC level	Staff	S 05

#### Findings

**Fig. 2:**
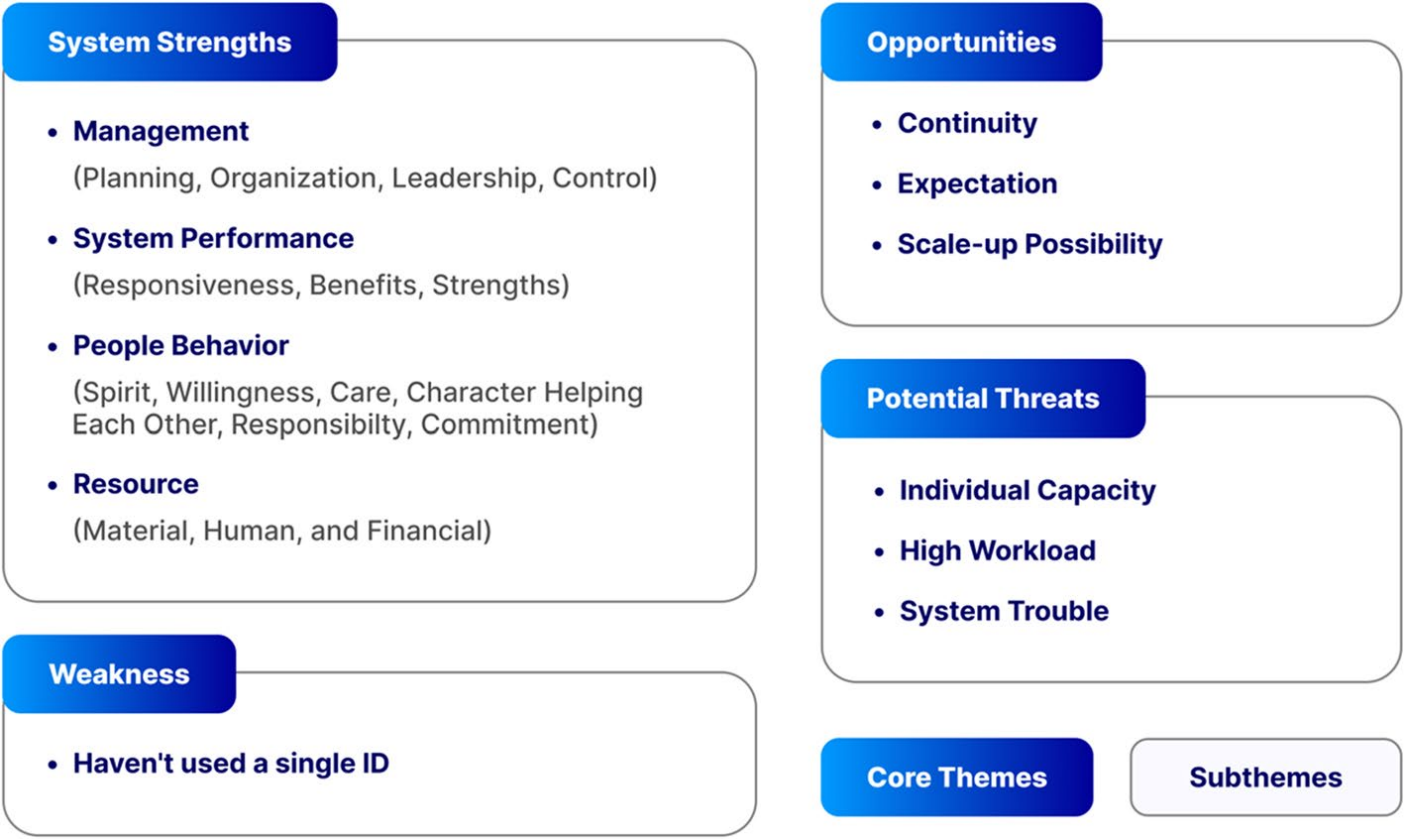
Strengths, potential threats, and opportunities for scaling-up

Findings from the study are organized and presented across the three core themes that emerged from the qualitative analysis, notably system strengths, potential threats, and opportunities for scale-up. However, data from qualitative and quantitative data fed into the analysis of these core themes to cross-validate the findings (Fig. 2. Detailed findings from the survey are presented in Table Supplement 1.

#### System’s strengths

Factors contributing to the success of SIMUNDU include management, system performance, people’s behavior, and resources.

### Management

SIMUNDU arose due to concerns from the DIY Health Office immunization section around data quality, notably the need to address data inaccuracy, duplicate or missing data and a lack of timely data, and the need for quality data to support follow-up and appropriate planning. The need for SIMUNDU arose from these challenges and needs.

“To our knowledge, [SIMUNDU development] started with a problem: estimates of the target population varied depending on the data source.” (M02)

“Yes, I think [SIMUNDU management team] started to tire of managing a large volume of data with dubious validity. They need to know the situation in each district.” (M04)

Effective management of SIMUNDU from development to implementation was highlighted as an essential determinant of its success across the critical functions of planning, organization, leadership, and control.

Careful planning was ensured at each stage of the development and implementation of SIMUNDU. These stages included developing an initial business plan, providing training on and socialization to SIMUNDU, and developing a staff replacement plan to respond to turnover or retirement of staff in charge of operating the system or entering data. The parties involved in planning included the Head of the Disease Prevention and Control Department, IT personnel, and, from the DIY Health Office, immunization program staff.

**Fig. 3:**
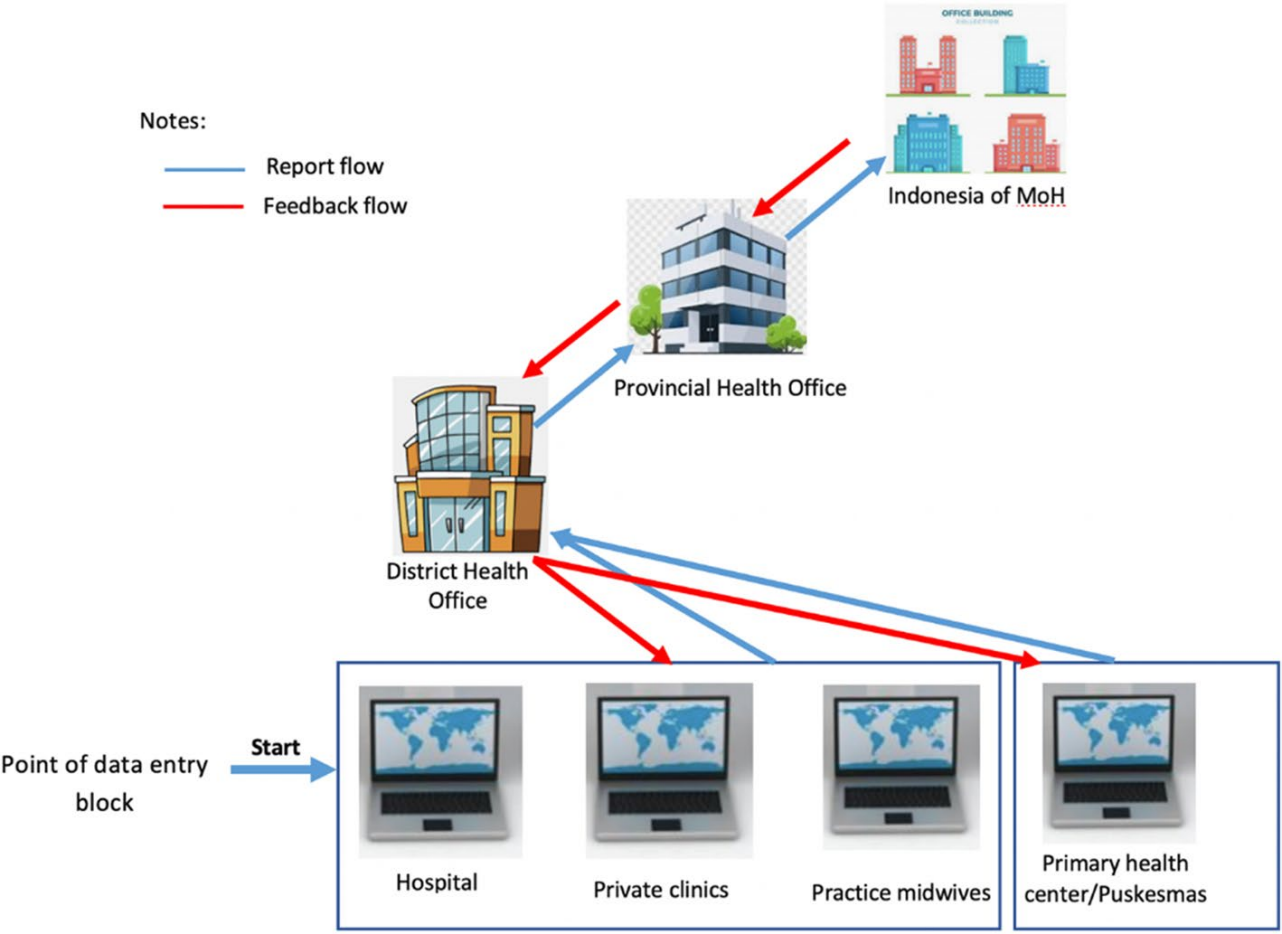
Visual organizing framework of SIMUNDU – DIY province, Indonesia

Organization – the organization of SIMUNDU is carried out at several levels. The top level is the DIY Health Office, the second level is the district/city health office, and the third level is health facilities (Fig. 3). A third party was also involved in developing the system interface.

At the beginning of the development of SIMUNDU, essential functions included database administrators, interface designers, and server administrators, and their interplay facilitated the system’s smooth operation. Training specific to SIMUNDU was integrated with other training, typically immunization-related. This enabled us to share resources with other programs, thus ensuring viability. The training was delivered in the district/city health office: 87.6%, 72%, and 75% of survey respondents from PHC, UPS, and DHO/CHO, respectively, participated in in-house training. Training typically consisted of short sessions and included practice on the trainee's device in operating the system in both online and offline mode. Informants indicated that day-to-day operations were carried out autonomously by the staff through flexibly adjusting their work to protect the time to enter the data. This seemed to work effectively.

Leadership – the success of SIMUNDU implementation is arguably related to strong leadership. Informants noted that managers played a crucial role in bridging the needs of the immunization program with the system design, closely monitoring the initial implementation process, and creating an enabling environment.

“I try to combine supporting and managing and monitoring the people involved. Currently, I monitor whether [SIMUNDU] can run optimally as our users are health facilities. I also monitor program development and the system's output.” (M01).

“[SIMUNDU] was born from program managers, primary health centers, districts, and DIY health offices wanting to build systems together. We – DIY Health Office – give them motivation in every meeting.” (M03).

“I see that [management] is very good at networking. Staff data entries in the field always indicated that these people are very kind.” (M02)

The role of IT workers in developing SIMUNDU was also significant. They helped develop the system and facilitated correct entries by entry clerk whenever technical issues arose. IT workers also helped resolve inconsistencies in data records. Acknowledgment of staff efforts was also important to maintain motivation and buy-in.

“In the early days of SIMUNDU’s development, the system was challenging to operate, as it wasn’t as stable as it is now. I praise the enthusiasm and dedication of the users.” (M01)

The control function – consisting of quality assurance management – was critical to avoid data duplication or missing entries and ultimately ensure data quality. This process was not regulated by specific standard operating procedures but was addressed during training and monitored monthly. In addition, the DIY Health Office provided negative incentives to health facilities that were not submitting complete records and provided regular feedback from monitoring and evaluation exercises.

Specifically, 94.2%, 100%, and 100% of survey respondents in PHC, UPS, and DHO, respectively, reported that their work had been subject to monitoring. More than half of the respondents in PHC and UPS facilities had been observed by supervisors while performing data entry at least once over the past year. At the PHC level, 48.3% of survey respondents had been subject to monitoring from the district/city office’s team, and 45.7% received monitoring from DIY Health Office staff. Conversely, 40% of respondents from UPS facilities were monitored by PHC staff. Almost all survey respondents reported receiving feedback from the monitoring, mainly from the district/city and DIY health offices. Forty percent of respondents from UPS facilities reported receiving feedback from PHC. Immunization coordinators from the district/city health offices received feedback from the DIY health offices.

“In a [evaluation] meeting, the DIY Health Office or District Health Office showed the progress of our data entry – correct or not, proper or not.” (M02)

It is worth noting that DIY province is quite a small geographic area. Because it consists of only five districts and one city, this province is relatively easy to monitor across all phases, from planning through monitoring and evaluation.

### System performance

While SIMUNDU predominantly contains individual-level immunization records, it also serves as a source for aggregation and can synergize with other information systems. Notably, SIMUNDU can link to the DHIS2 and generate immunization-specific reports as per the Ministry of Health’s requirements. These reports are sent to the upper levels automatically if SIMUNDU is operated online or submitted via email if SIMUNDU is operated offline. This functionality has had an essential role in ensuring the acceptability and adoption of the system.

Informants noted how transitioning from paper-based tools to an electronic system made data entry easier and reduced errors. SIMUNDU also facilitated the implementation of protocols for data storage and security. It enabled follow-up and defaulter tracking. Finally, integration with the DHIS2 meant reduced workload for the staff.

“We can track children who may have received vaccinations in different locations faster. For example, when the first dose of a vaccine is given in Bantul and the second one in Yogyakarta, the record can be linked within SIMUNDU.” (M01).

“SIMUNDU makes detecting what data and vaccinations are missing easier since we enter data from the children’s birth through the end of the immunization schedule. So, we will know where they miss any vaccine.” (S03).

“The benefit of using SIMUNDU is first: we know the situation of immunizations more accurately….so our vaccine forecasting is more accurate …. and our budget, staff, facilities can be more effective and efficient in providing services.” (S05).

“Colleagues from the mother and child health (KIA) program enter the data via the KIA "Sembada." So, these data will appear automatically in SIMUNDU because the two systems are connected.” (S01).

SIMUNDU is user-friendly and can be flexibly operated offline or online, allowing the responsible staff to maintain data entry irrespective of connectivity; 82.3%, 96%, and 100% of survey respondents from PHC, UPS, and DHO, respectively, reported operating SIMUNDU online.

### People’s behavior

The interview showed that staff commitment was critical for the successful implementation of SIMUNDU, as indicated by their willingness to work overtime and bring home the data to enter into the system.

“I take it [the data] home too, for example, after immunization sessions – in my clinic, immunization runs four times per month, every week. So, when the session is finished, we can take the data home, [and] do the entry at home while relaxing.” (S03).

The interviews confirmed this dedication, which spoke to a societal culture of helping others and responsibility and commitment to the team. This contributed to shaping an environment where people approach SIMUNDU as a shared responsibility and a collective endeavor. Informants also noted the high motivation of dedicated staff.

“That's all; we cannot judge by money [people’s kindness, culture, and behavior]; explaining how good people are in Yogyakarta is essential. I was in another place before, and could not find people's kindness like in Yogyakarta – different characters.” (M02).

“The second thing is that we need human resources that are concerned about, and have a love for, data; otherwise, even if we have a good system, it will amount to nothing without good human resources. But good implementation will come more easily when people are concerned about data.” (M04).

### Resources: material, human, and financial

Infrastructure and equipment emerged as critical factors in introducing and sustaining SIMUNDU implementation. Some desktops were explicitly allocated to the immunization program, and some had to be shared with other staff. Other data entry officers reported using their laptop or smartphone (36.3% of survey respondents from PHC). In UPS facilities, 40.7% reported using office desktops; in the DHO, more than half of the respondents said they used an office-supplied laptop. The majority of respondents – regardless of the type of facility – said their current device was sufficient to perform their work on SIMUNDU. In terms of connectivity, 64.6% of PHC survey respondents and 67.7% of UPS’s reported operating SIMUNDU online, relying on the office’s Internet connection.

Management of financial resources was also crucial. According to the key informants, no special funds were allocated to SIMUNDU in the initial stages. Resources were leveraged through sharing activities – e.g., monitoring visits or transportation – with other programs, thus allowing cost efficiencies. Integration with other programs proved critical to ensuring sustainability.

“SIMUNDU's budget comes from the state budget known as Anggaran Pendapatan dan Belanja Negara (APBN). Every year the APBN allocates funding envelopes for immunization to DIY and other provinces, where the budget is apportioned across the program [not an explicitly written budget for SIMUNDU].” (M02).

Human resources are critical to the operation of SIMUNDU. According to the interviews, SIMUNDU data entry clerks must have patience, work carefully and not rush, be interested in data, be responsible, and have basic computer skills in word processing and spreadsheet software tools such as Microsoft Word and Excel, respectively. As shown by the survey, the large majority of SIMUNDU-operating staff were educated: At least 80% of data entry clerks in both PHC and UPS facilities have secondary education (> 80%), while at the managerial level (DHO), 75% of respondents have a bachelor’s degree (see Table 2). However, 19.4% and 9.1% of respondents from PHC and UPS facilities have low computer literacy.

Various data entry clerks looked for strategies to resolve the obstacles they encountered when entering data into SIMUNDU. Based on the interviews, some clerks furthered their computer skills by taking private computer classes. Others learned from colleagues in their offices, or reached out for help to the district person in charge. To deal with the accumulation of data needing to be entered in SIMUNDU, staff would sometimes work at home after office hours, as their busy schedule at work did not allow time for data entry.

“If we found obstacles, we asked people in charge in PHC – asking for a solution or sharing by WhatsApp – or sometimes I asked the IT person in the DIY Health Office.” (S03)

### Potential threats

As of today, SIMUNDU can be said to be a successful experience. However, some obstacles were encountered and addressed during implementation. Potential system sustaining includes individual capacity, technical or system issues, and high workload. Staff computer literacy was identified as one of the main sustainability challenges. Internet connectivity was another obstacle, as a good network did not support all health facilities. The survey shows that 64.6% and 67.7% of PHC and UPS staff, respectively, used the office Internet, while others had to rely on their home Internet.

Further incomplete and inconsistent records – such as a different child's date of birth or name spelling across relevant entries – make it challenging to consistently record immunization information. These challenges have arisen during implementation and were promptly addressed. Yet, they had an impact on staff who were already juggling busy schedules in the office, causing delays in data entry. As shown by the survey, almost all respondents said they had other responsibilities besides operating SIMUNDU – notably 97.3%, 88%, and 100% of participants from PHC, UPS and district and city offices, respectively.

### Weakness

Some informants said that SIMUNDU assisted in their daily work, but they also reported that sometimes they needed more time to find the children's names on the next visit. This is because SIMUNDU data entry did not use a single national ID that could be valid anywhere. As a result, when a name input error occurs, the officer will need time to check the name with the child's parents or the manual register.

“Sometimes, there was an incorrect name during the data entry; for example, Dita was written as Dieta. So, it is difficult for us to find them. If that happens, we must look back at the register or medical record data.” (S04).

“I experienced difficulty entering data in SIMUNDU when a new patient came from another health facility to us. It was challenging to find their record on SIMUNDU.” (S05).

### Opportunities

Informants appreciated SIMUNDU as an excellent system to manage immunization data. SIMUNDU has become necessary for program managers and policymakers; it allows them to monitor coverage and can help inform planning and programming. Currently, SIMUNDU is stable, thus it is easier to manage than when it was in the development phase. It is also viable and no longer requires heavy reliance on the core workforce that started the system. The hopes expressed by data entry clerks in the interviews are that SIMUNDU is easier to operate and system errors are less frequent. Informants also stressed the need for refresher training to ensure that knowledge and practice of the system is not lost.

“In my opinion, SIMUNDU is the best program in DIY, a collaboration between program managers and IT. It will continue to be implemented because it is a necessity. It has been stably used for more than five years, meaning this is needed.” (M01).

“If I have the tool, in this case SIMUNDU, when it is stable, whoever will be able to run it, I am sure that anyone can operate it. It means that it doesn't matter if we have people shifting [jobs].” (M01).

“In the future, if SIMUNDU is still used, other reports are not necessary. Now we have two different reports: SIMUNDU and stock card of vaccine – each stands alone and needs a separate report.” (S05).

Based on the key informants’ interviews, SIMUNDU is likely to be developed further or expanded to other provinces. The DIY Health Office is open to supporting other provinces interested in introducing the system – for instance, through the lending staff for training and orientation. However, informants advised that a successful introduction requires a strong commitment from staff and management.

## Discussion

Robust health information systems (HIS) are essential components of robust health systems [13]. At the most basic level, immunization registries are systems that collect and report individual-level vaccine administration record data, thus facilitating individual follow-up of vaccination status. Registries also allow for the monitoring of vaccination coverage and enable analysis of AEFIs and surveillance data to inform the design of coverage interventions and outbreak investigations. When an electronic registry has interoperability with other electronic systems – such as in the case of SIMUNDU – it is considered an immunization information system (IIS) [14]. This paper presents lessons learned from DIY’s experience of implementing an IIS.

DIY is the only province in Indonesia – out of 34 – that uses an IIS. This work has shed light on the strengths of, and underlying barriers to, implementing an IIS in this context. The objective of this study was to draw lessons that inform sustainable scaling-up in other provinces and possibly at the national level. This study highlighted individual capacity, technical or system issues, and high workload as the major barriers to sustainability. Conversely, management, system performance, people’s behavior, and available resources emerged as the main determinants of SIMUNDU’s successful implementation – notably in improving acceptability, implementation costs, and adoption of this innovation [15].

Despite several obstacles encountered during the implementation of SIMUNDU, this study showed that this innovation was well accepted by key stakeholders. On the one hand, data entry clerks noted that the system is relatively user-friendly and makes it possible to organize the data better and enhance its quality. On the other hand, managers noted the benefits this innovation brought about, namely in the potential for cohort data to support planning and monitoring and ultimately improve immunization coverage.

Effective management – across planning, organization, leadership, and control functions – is a crucial reason why SIMUNDU has been viable for over five years. Managers use their control to encourage the beliefs and actions of the staff with a dedicated and robust managerial process [16]. SIMUNDU was born from the need for credible data to assist in carrying out DIY Health Office duties at the managerial and operational levels. At the managerial level, the disease prevention and control department and the IT department collaborated in designing a system that intended users readily accepted. Immunization officers and IT programmers played a central role from the early stages of development through implementation with effective coordination and communication. They were helped in this task, with the full support of their respective superiors.

SIMUNDU is cost-effective in several ways. During the introductory period of its implementation, immunization programmers, IT officers, and other staff assisted in introducing SIMUNDU in all districts in the province. This was done by integrating some of the activities across programs, thus building efficiency in terms of time and costs for both managers and staff. Sharing resources across programs was critical in the first years of building sustainability. Additionally, maintaining SIMUNDU does not incur high costs because the DIY Health Office has developed the system and thus possesses in-house technical skills. The IT department has the capacity to monitor and improve processes and tailor them to user needs without much additional cost.

A good program without good leadership could fail in its implementation, and even if it was initially successful, it might not be sustainable [17]. In the context of SIMUNDU, leadership and effective management support facilitated the program's adoption. The uptake of the new system was good and all health facilities providing immunization services have successfully transitioned to SIMUNDU. The strong network of the prominent persons in charge of SIMUNDU also facilitated the adoption. Good communication, care, and attention to staff concern positively affected staff performance. They felt that they were well supported and treated kindly, and this helped them carry out their work joyfully. According to several informants, the DIY immunization program manager’s leadership played an essential role in this effect.

The monitoring and evaluation mechanisms of SIMUNDU were also important. Preferred monitoring and evaluation activities include monthly reports and staff discussions during site monitoring visits. The immunization program manager suggested this approach to maintain data quality and ensure the system’s sustainability. These chosen mechanisms allow program managers to assess the actual practice in the field and the challenges faced to inform decisions about the follow-up actions to be taken. These processes supported the ongoing development of, and learning from, SIMUNDU as a tool for data collection, analysis, and visualization, as well as the benefits for managers in carrying out monitoring and evaluation. The same sentiment was reflected in previous research undertaken in India [18].

Human resources are a key determinant of the successful implementation of any HIS [19]. People's behavior affects how the system works, develops, and survives [20, 21]. In the case of SIMUNDU, implementation was facilitated by a culture of care, established networks, and a positive attitude towards data on the part of both the program manager and the IT team. From the staff's point of view, the local culture of helping each other and doing their job correctly and responsibly translated into staff carrying out their duties with enthusiasm and great commitment. Although facilities, funding, and human resources were limited, the individuals involved were highly motivated and supportive.

Despite the many strengths of SIMUNDU, some obstacles may potentially challenge its sustainability in the long term. These obstacles can be divided into human variables and technical variables. In terms of human variables, unequal capacity distribution at the operational level can result in differing levels of data quality across facilities and districts. Staff workload is another challenge that needs addressing, as their willingness to work overtime is not a sustainable strategy. Technical problems were another obstacle during the introduction of SIMUNDU, but qualified technicians/developers were able to solve these issues. During our research, we recognized the weakness of SIMUNDU that it had not used the person number as a unique (single) code (ID) in data entry. This impacts on the challenge of finding a person when the previous entry was inaccurate. The in absence SIMUNDU single ID also affects SIMUNDU's inability to synchronize with other health programs that use a person's number as a unique code. However, this weakness can be seen as room for improvement for SIMUNDU shortly. Another thing that needs to be considered for other regions that want to implement SIMUNDU, so far SIMUNDU is implemented in DIY province, which consists of five districts/cities with relatively easy regional accessibility. For areas with more difficult access, the commitment of the leadership and subordinates is the key to successful implementation.

## Conclusion

SIMUNDU is a promising innovation for the entire country, beyond DIY. There is agreement about the potential for scaling-up of this IIS to other provinces. Experience of implementing this system in DIY over the past five years has shown that the benefits outweigh the challenges, and SIMUNDU has grown into a robust yet user-friendly system. Regular training for dedicated staff to strengthen their capacity as the system evolves and is updated, and a plan for anticipating and responding to staff turnover, have proven critical strategies towards sustainability. SIMUNDU’s success also rests on remarkable leadership, both in creating and enabling a supportive environment and pursuing integration with other programs to share limited resources.

### Recommendations

This study’s recommendations address three different stakeholder groups: the DIY Health Office, the national government, and researchers. First, to ensure continuity and sustainability and reduce the system's dependency on a particular person or party, SIMUNDU management and maintenance should be managed by people who have competency and interest in a good reporting system. Furthermore, a human resources plan should be developed in preparation for SIMUNDU rollout in other provinces or at the national level; this is necessary to avoid vacancies when DIY province staff are seconded to other areas for mentoring support. Second, the fact that SIMUNDU emerged from an actual need for immunization program implementers and saw these at the front line of its development and implementation positively impacted its feasibility and viability. This suggests that the approach to scaling up SIMUNDU should be stepwise, taking into consideration each region’s specific characteristics and needs. To this effect, a readiness map and a timeline may be developed to roll out SIMUNDU in a particular region. Third, further research is needed to assess the impact of SIMUNDU on immunization coverage. Based on our conversations with stakeholders, it would be particularly relevant to focus on a low-performing region and observe the impact over a two- to three-year time window.

### Study limitations

The empirical results reported herein should be considered in light of limitations. First, the results of the quantitative study must be considered in view of the limited sample size, particularly for UPS health facilities. However, given the top-down immunization program and the characteristics of UPS, which will not be significantly different from each other, the results of this study are still valid and relevant to the existing condition. In qualitative research that aims to explore, caution is needed in interpreting the interview results. There is still a need for in-depth studies with different approaches, such as focus group discussions, to confirm the results.

## Supplementary Information

**Additional file 1.**

## Data Availability

The data sets generated and/or analyzed for this study can be requested from the corresponding author.

## Abbreviations

AEFI: Adverse Events Following Immunization
APBN: Anggaran Pendapatan dan Belanja Negara (State Budget)
CHO: City Health Office
COVID-19: Coronavirus Disease 2019
DHIS2: District Health Information System 2
DHO: District Health Office
DIY: Daerah Istimewa Yogyakarta (Special Region of Yogyakarta)
DT: Diphtheria Toxoid
HIS: Health Information System
HPV: Human papillomavirus
ID: Identity
IIS: Immunization Information System
IT: Information Technology
KIA: Kesehatan Ibu dan Anak (Maternal and Child Health)
KOMDAT KESMAS: Komunikasi Data Kesehatan Masyarakat (Public Health Data Communication)
MR: Measles-Rubella
PHC: Primary Health Centers
PHO: Provincial Health Office
PISPK: Program Indonesia Sehat dengan Pendekatan Keluarga (Healthy Indonesia Program with Family Approach)
PWS: Pemantauan Wilayah Setempat (Local Area Monitoring)
SIHA: HIV AIDS Information System
SIKDA: Regional Health Information System
SIMUNDU: Integrated Immunization Information System
SITT: Integrated Tuberculosis Information System
TD: Tetanus-Diphtheria
UPS: Unit Pelayanan Swasta (Private Service Unit)
