# Supplementary material for: Introduction and implementation of an immunization information system in the Indonesian province of Daerah Istimewa Yogyakarta: lessons for scaling-up
Source: BMC Health Serv Res. 2023 Jan 5;23:12. doi: 10.1186/s12913-022-08910-6 (PMC9815049; doi:10.1186/s12913-022-08910-6)
Supplement: Supplementary file 1 — Additional file 1. [file 12913_2022_8910_MOESM1_ESM.docx]

**Table 1**. Respondent response duriWehang the survey

| **Questions** | **PHC (n= 113)**  **n (%)** | **UPS (n=25)**  **n (%)** | **DHO/CHO (n= 4)**  **n (%)** |
| --- | --- | --- | --- |
| Among the two systems – Offline and Online – which one do you prefer?  ONLINE  OFFLINE | 93 (82.3)  20 (17,7) | 24 (96.0)  1 (4.0) | 4 (100)  0 (0.0) |
| Do you carry out any other work/duties besides SIMUNDU?  No  Yes | 3 (2,7)  110 (97,3) | 3 (12.0)  22 (88.0) | 0 (0.0)  4 (100) |
| Who is the main person in charge of doing data entry to SIMUNDU in your office?  Myself  Other | 96 (85.0)  17 (15.0) | 18 (72.0)  7 (28.0) | 3 (75.0)  1 (25.0) |
| How long have you been in charge of entering immunization data using SIMUNDU?  <1 year  1-2 year  2-3 year  3-4 year  >4 year | 8 (7.1)  7 (6.2)  16 (14.2)  17 (15.0)  65 (57.5) | 5 (20.0)  15 (60.0)  1 (4.0)  1 (4.0)  3 (12.0) | * |
| How long have you been in charge of managing SIMUNDU?  <1 year  1-2 year  2-3 year  3-4 year  >4 year | * | * | 1 (25.0)  0 (0.0)  0 (0.0)  0 (0.0)  3 (75.0) |
| 23. Of the several items below, which ones you can operate to support work at SIMUNDU?  Excel spreadsheet  Extract file  Export-import file  Email/browsing  Other  *Respondent allows selecting more than one response.* | 61 (23.6)  42 (16.3)  58 (22.5)  92 (35.7)  5 (1.9) | 14 (32.6)  4 (9.3)  6 (14.0)  18 (41.9)  1 (2.3) | 1 (20.0)  1 (20.0)  1 (20.0)  2 (40.0)  0 (0.0) |
| **Barrier perception** |  |  |  |
| Have you ever had difficulty operating SIMUNDU?  Yes  No | 93 (82.3)  20 (17.7) | 16 (64.0)  9 (36.0) | 2 (50.0)  2 (50.0) |
| When experienced with difficulties in operating SIMUNDU, with whom you discuss to ask solutions?  Puskesmas / PHC  District health office  DIY health office  Other (staff in other health facilities)  *Respondent allows selecting more than one response.* | 17 (9.6)  73 (41.0)  66 (37.1)  22 (12.4) | 13 (56.6)  6 (26.1)  2 (8.7)  2 (8.7) | 0 (0.0)  0 (0.0)  2 (100)  0 (0.0) |
| Are you satisfied with the follow-up taken from the results of the consultation?  No  Yes | 1 (1.1)  92 (98.9) | 0 (0.0)  16 (100) | 0 (0.0)  4 (100) |
| **Report Timeliness** |  |  |  |
| In SIMUNDU OFFLINE that has been running so far, have you sent the report according to the specified date?  No  Yes  I’m operating SIMUNDU online. | 17 (15.0)  87 (77.0)  9 (8.0) | 4 (16.0)  21 (84.0)  0 | 1 (25.0)  3 (75.0)  0 (0.0) |
| On the SIMUNDU OFFLINE. Did you experience any obstacles in the SIMUNDU data entry on time?  No  Yes | 67 (59.3)  46 (40.7) | 16 (64.0)  9 (36.0) | * |
| On the SIMUNDU OFFLINE. Did you have any obstacles in reporting SIMUNDU data on time?  Difficulties on the export file  Difficulties on email or sending files  Difficulties in the extracted file  Other | 10 (17.2)  13 (22.4)  9 (15.5)  26 (44.8) | 4 (36.4)  0  2 (18.2)  5 (45.5) | * |
| Pada SIMUNDU ONLINE, when do you input your baby/toddler data into SIMUNDU  The same day after the service is finished  <1 week after service  One week - 1 month after service  > 1 month after service | 37 (25.7)  50 (34.7)  48 (33.3)  9 (6.3) | 4 (16.0)  10 (40.0)  10 (40.0)  1 (4.0) | * |
| In the ONLINE system, do you have any obstacles in entering data in SIMUNDU timely?  No  Yes | 52 (46.0)  61 (54.0) | 14 (56.0)  11 (44.0) | * |
| In OFFLINE systems – in 5 scales. How many do you assess the timeliness of the reports you have provided so far?  1  2  3  4  5 | 0 (0.0)  3 (2.7)  36 (31.9)  60 (53.1)  14 (12.4) | 1 (4.0)  1 (4.0)  9 (36.0)  12 (48.0)  1 (8.0) | 0  0  0  3 (75.0)  1 (25.0) |
| **Data Accuracy** |  |  |  |
| Have you ever found the data entered at SIMUNDU to be different from the data in the immunization service register?  No  Yes | 29 (25.7)  84 (74.3) | 14 (56.0)  11 (44.0) | * |
| **Data verification** |  |  |  |
| Have you ever verified the data between the data in SIMUNDU and the data in the immunization service register?  No  Yes | 5 (4.4)  108 (95.6) | 8 (32.0)  17 (68.0) | * |
| When is data verification done?  Monthly  Bimonthly  Three months  Semester  Other | 42 (38.9)  4 (3.7)  23 (21.3)  17 (15.7)  22 (20.4) | 10 (58.8)  2 (11.8)  1 (5.9)  1 (5.9)  3 (17.6) | * |
| **Data completeness** |  |  |  |
| According to you, are there a lot of menus/items to input into SIMUNDU?  No  Yes | 51 (45.1)  62 (54.9) | 18 (72.0)  7 (28.0) | * |
| In your opinion, is the completeness of the menu/item entries in SIMUNDU important?  No  Yes | 1 (0.9)  112 (99.1) | 2 (8.0)  23 (92.0) | * |
| **SIMUNDU accessibility** |  |  |  |
| Do you agree with the statement that "SIMUNDU is easy to operate?  Agree  Disagree | 108 (95.6)  5 (4.4) | 23 (92.0)  2 (8.0) | 4 (100)  0 (0.0) |
| Did you analyse the SIMUNDU data?  No  Yes | 26 (23.0)  88 (77.0) | * | 0 (0.0)  (4 )100) |
| **Over/Under reporting** |  |  |  |
| Do you have any experience finding data on children/babies in the Immunization Service Register that are not reported to SIMUNDU?  No  Yes | 36 (31.9)  77 (68.1) | 13 (52.0)  12 (48.0) | 0 (0.0)  4 (100) |
| Do you have the experience of finding children data in the Immunization Service Register that entry with more than one?  No  Yes | 38 (33.6)  75 (66.4) | 15 (60.0)  10 (40.0) | 1 (25.0)  3 (75.0) |
| According to you, does under or over-reporting have an impact on the achievements of the immunization program?  No  Yes | 5 (4.4)  108 (95.6) | 1 (4.0)  24 (96.0) | 1 (25.0)  3 (75.0) |
| **Facility and infrastructure** |  |  |  |
| What type of computer do you most use to enter data in SIMUNDU?  Private laptop  Laptop – office facility  PC – office facility  PC - private  Handphone  Other | 41 (36.3)  38 (33.6)  32 (28.3)  0  0  2 (1.8) | 4 (14.8)  4 (14.8)  11 (40.7)  0 (0.0)  7 (25.9)  1 (3.7) | 1 (25.0)  2 (50.0)  1 (25.0)  0 (0.0)  0 (0.0)  0 (0.0) |
| Does your current computer/handphone/laptop support your work on operating SIMUNDU?  No  Yes | 11 (9.7)  102 (90.3) | 0 (0.0)  25 (100) | 1 (25.0)  3 (75.0) |
| Where are your internet sources from?  None  Office facility (Wifi)  Data packages pay with their own money  Data packages paid by the office  Other  *Respondent allows selecting more than one response* | 0 (0.0)  102 (64.6)  48 (30.4)  1 (0.6)  7 (4.4) | 0 (0.0)  21 (67.7)  9 (29.0)  0  1 (3.2) | 3 (75.0)  1 (25.0)  0  0  0 |
| Is the internet facility that you use, suit your needs for data entry SIMUNDU?  No  Yes | 18 (15.9)  95 (84.1) | 2 (8.0)  23 (92.0) | 1 (25.0)  3 (75.0) |
| Where is the source of your electricity?  PLN  Genset  None  Other  *Respondent allows selecting more than one response.* | 114 (80.9)  27 (19.1)  0  0 | 25 (80.6)  6 (19.4)  0  0 | 4 (100)  0  0  0 |
| Do you have any problems with electricity during SIMUNDU entry?  No  Yes | 78 (69.0)  35 (31.0) | 24 (96.0)  1 (4.0) | 4 (100)  0 |
| From your side, what are the obstacles in SIMUNDU reporting?  It is difficult for data entry at SIMUNDU  Do not have time  Have another assignment  My computer skill is poor  Never received SIMUNDU training  Other  *Respondent allows selecting more than one response* | 7 (3.7)  18 (9.4)  95 (49.7)  37 (19.4)  11 (5.8)  23 (12.0) | 2 (6.1)  7 (21.2)  16 (48.5)  3 (9.1)  2 (6.1)  3 (9.1) | 0  1 (20.0)  2 (40.0)  0  1 (20.0)  1 (20.0) |
| **Managerial Process** |  |  |  |
| Do you know the purpose of SIMUNDU development in DIY?  No  Yes | 18 (15.9)  95 (84.1) | 6 (24.0)  19 (76.0) | 0  4 (100) |
| Have you ever participated in SIMUNDU in house training?  No  Yes | 14 (12.4)  99 (87.6) | 7 (28.0)  18 (72.0) | 1 (25.0)  3 (75.0) |
| When did you last take part in the SIMUNDU in house training?  < 1 year ago  > 1 year ago | 42 (42.4)  57 (57.6) | 11 (61.1)  7 (38.9) | 0  2 (100) |
| Which institution conducts SIMUNDU in house training, that you ever attended?  Puskesmas (PHC)  District/City health office  DIY health office  Other  *Respondent allows selecting more than one response* | 0 (0.0)  56 (38.9)  88 (61.1)  0 (0.0) | 2 (8.0)  15 (60.0)  7 (28.0)  1 (4.0) | 0  0  0  2 (100) |
| What training guides are used during training?  PPT  Word – hard copy  Word - soft file  Other | 80 (49.1)  32 (19.6)  36 (22.1)  15 (9.2) | 12 (56.1)  2 (9.5)  2 (9.5)  5 (23.8) | 2 (50.0)  1 (25.0)  1 (25.0)  0 |
| Have you ever been monitored and evaluated regarding SIMUNDU?  No  Yes | 10 (8.8)  103 (91.2) | 6 (24.0)  19 (76.0) | 0  4 (100) |
| In the last year (July 2019-July 2020), how many times monitoring and evaluation been conducted?  >2 times  One time  1-2 times | 17 (16.5)  57 (55.3)  29 (28.2) | 2 (10.5)  11 (57.9)  6 (31.6) | 2 (50.0)  1 (25.0)  1 (25.0) |
| Who did monitor and evaluation SIMUNDU on your place?  Puskesmas (PHC)  District/City health office  DIY health office  Other  *Respondent allows selecting more than one response* | 11 (7.0)  81 (51.3)  65 (41.1)  1 (0.6) | 11 (39.3)  8 (28.6)  8 (28.6)  2 (3.6) | 0  0  4 (100)  0 |
| Did you receive any feedback on the results of the SIMUNDU monitoring and evaluation?  No  Yes | 6 (5.8)  97 (94.2) | 0 (0.0)  19 (100) | 0  4 (100) |
| Who gave feedback on the M&E results?  Puskesmas (PHC)  District/City health office  DIY health office  Other  *Respondent allows selecting more than one response* | 7 (4.6)  73 (48.3)  69 (45.7)  2 (1.3) | 10 (40.0)  8 (32.0)  6 (24.0)  1 (4.0) | 0  0  4 (100)  0 |
| In the last year ((July 2019 - July 2020), have you ever monitored the health facility under your supervision?  Yes  No | * | * | 4 (100)  0 |
| Have you ever participated in the dissemination of M&E results as well as updating knowledge?  No  Yes | 28 (24.8)  85 (75.2) | 16 (64.0)  9 (36.0) | 0  4 (100) |
| Who is organizing the dissemination of M&E results as well as updating the knowledge?  Puskesmas (PHC)  District/City health office  DIY health office  Other  *Respondent allows selecting more than one response* | 3 (2.5)  53 (44.2)  63 (52.5)  1 (0.8) | 0 (0.0)  6 (60.0)  4 (40.0)  0 (0.0) | 0  1 (25.0)  3 (75.0)  0 |

*data not applicable
